# Substrate Stiffness Determines the Establishment of Apical-Basal Polarization in Renal Epithelial Cells but Not in Tubuloid-Derived Cells

**DOI:** 10.3389/fbioe.2022.820930

**Published:** 2022-03-01

**Authors:** Maria J. Hagelaars, Fjodor A. Yousef Yengej, Marianne C. Verhaar, Maarten B. Rookmaaker, Sandra Loerakker, Carlijn V. C. Bouten

**Affiliations:** ^1^ Eindhoven University of Technology, Department of Biomedical Engineering, Eindhoven, Netherlands; ^2^ Institute for Complex Molecular Systems (ICMS), Eindhoven, Netherlands; ^3^ Hubrecht Institute, Royal Netherlands Academy of Arts and Sciences, Utrecht, Netherlands; ^4^ Department of Nephrology and Hypertension, University Medical Center Utrecht, Utrecht, Netherlands

**Keywords:** cell polarization, substrate stiffness, mechanosensing, madin darby canine kidney cells, tubuloids

## Abstract

Mechanical guidance of tissue morphogenesis is an emerging method of regenerative medicine that can be employed to steer functional kidney architecture for the purpose of bioartificial kidney design or renal tissue engineering strategies. In kidney morphogenesis, apical-basal polarization of renal epithelial cells is paramount for tubule formation and subsequent tissue functions like excretion and resorption. In kidney epithelium, polarization is initiated by integrin-mediated cell-matrix adhesion at the cell membrane. Cellular mechanobiology research has indicated that this integrin-mediated adhesion is responsive to matrix stiffness, raising the possibility to use matrix stiffness as a handle to steer cell polarization. Herein, we evaluate apical-basal polarization in response to 2D substates of different stiffness (1, 10, 50 kPa and glass) in Madin Darby Canine Kidney cells (MDCKs), a classic canine-derived cell model of epithelial polarization, and in tubuloid-derived cells, established from human primary cells derived from adult kidney tissue. Our results show that sub-physiological (1 kPa) substrate stiffness with low integrin-based adhesion induces polarization in MDCKs, while MDCKs on supraphysiological (>10 kPa) stiffness remain unpolarized. Inhibition of integrin, indeed, allows for polarization on the supraphysiological substrates, suggesting that increased cellular adhesion on stiff substrates opposes polarization. In contrast, tubuloid-derived cells do not establish apical-basal polarization on 2D substrates, irrespective of substrate stiffness, despite their ability to polarize in 3D environments. Further analysis implies that the 2D cultured tubuloid-derived cells have a diminished mechanosensitive capacity when presented with different substrate stiffnesses due to immature focal adhesions and the absence of a connection between focal adhesions and the cytoskeleton. Overall, this study demonstrates that apical-basal polarization is a complex process, where cell type, the extracellular environment, and both the mechanical and chemical aspects in cell-matrix interactions performed by integrins play a role.

## 1 Introduction

Engineered tissues are developed to replace, restore, or enhance the biologic function of damaged tissues or organs. The mammalian kidney offers a major challenge for tissue engineers in terms of creating functional tissue architecture, as the kidney consists of millions of tubular structures that are interconnected to form a functional waste disposal system. Additionally, these tubular structures are segmented and consist of a heterogeneous pool of epithelial cells with highly specified functions for filtration and transport (O’Callaghan, 2016). The mechanical guidance of tissue morphogenesis is an emerging strategy to create functional kidney tissue architecture for the purpose of tubule tissue engineering, as well as for the bio-artificial kidney, where renal epithelial cells are seeded on two-dimensional (2D) biomaterial substrates to allow excretion and resorption.

Central to kidney morphogenesis is epithelial polarization (Jewett and Prekeris, 2018). Epithelial cell polarity is characterized by the asymmetric distribution of polarity proteins to separate apical and basal poles of the cells (Bryant and Mostov, 2008). This intrinsic asymmetry within a collective of epithelial cells is crucial for vectorial transport of intracellular vesicles containing fluid and apical proteins towards the apical membrane initiating site where a luminal space surrounded by these cells can be created *de novo* (Macara, 2004; Mellman and Nelson, 2008; Sigurbjörnsdóttir et al., 2014) The development of tubular structures with a central lumen surrounded by leak-tight polarized epithelium is paramount for key renal functions, including active and selective secretion and reabsorption of waste products and useful substances, respectively, (Gullans et al., 1996; Mellman and Nelson, 2008; Kocgozlu et al., 2016).

Earlier cell biology research suggests that the orientation of apical-basal polarity is initiated by integrin mediated cell-matrix adhesion at the basal site of renal epithelial cells (Wang et al., 1990a; Wang et al., 1990b; Ojakian and Schwimmer, 1994; Yu et al., 2005; Akhtar and Streuli, 2013). Upon adhesion, the subsequent signaling cascade leads to the formation of the apical surface facing opposite from the basal site (Bryant et al., 2010; Akhtar and Streuli, 2013). Concurrently, the cytoskeleton and the membrane-trafficking machinery organize asymmetrically. Cytoskeletal filaments, such as actin and microtubules, are inherently asymmetric polar filaments with two distinct ends. Actin polymerization is activated at both the basal membrane and the apical membrane creating a distinct basal and apical cortex, respectively. Inhibition of β1-integrins in Madin Darby Canine Kidney cells (MDCKs) has been demonstrated to prevent polarization in three dimensional (3D) *in vitro* cultures (Ojakian and Schwimmer, 1994; Yu et al., 2005).

Next to their role in biochemical signaling, integrins have been identified as one of the most important transmembrane receptors for interpreting physical and mechanical signals from the ECM in all cell types (Yeung et al., 2005; Lock et al., 2008). Integrin binding to the ECM results in the recruitment of proteins (e.g., talin, vinculin, zyxin) that together form larger focal adhesion (FA) complexes. The FA complexes are connected to the nucleus via the actin cytoskeleton, forming the mechanotransduction pathway. It has been well established that a rise in matrix rigidity of a 2D substrate or a 3D environment leads to increased activation, clustering and maturation of integrin complexes in adherent cells, which respond to the change in stiffness by changing their cytoskeletal organization, tension and cell morphology (Bershadsky et al., 2003; Yeung et al., 2005; Mekhdjian et al., 2017). Combining these two observations opens up the question if matrix stiffness can be used to control epithelial polarization for the purpose of tissue engineering or the development of the bio-artificial kidney.

Here, we investigate if and how 2D substrate stiffness can be used to steer apical-basal polarization in renal epithelial cells. First, we examined the effect of substrate stiffness on apical-basal polarization in MDCKs, a classic canine renal *in vitro* model commonly used in apical-basal polarization research. In view of our intention to apply the insights resulting from this study in the bio-artificial kidney or tissue engineering, we next examined the effect of substrate stiffness on apical-basal polarization in human kidney tubuloid-derived cells as a physiological model of the human renal epithelium. Human tubuloid culture allows for long-term propagation of donor-specific primary kidney epithelium without requiring immortalization or genetic modification. The tubuloids consist of a heterogeneous pool of epithelial cells from the proximal tubule, loop of Henle, distal tubule and collecting duct, as shown using bulk and single cell transcriptomics, immunofluorescence, and functional assays (Schutgens et al., 2019). The heterogeneity was reported to be limited over time in culture and between different donors and was therefore not expected to interfere with the reported results (Schutgens et al., 2019; Gijzen et al., 2021; Jamalpoor et al., 2021).

Both cell types were cultured on 2D collagen-I coated polyacrylamide (PAA) substrates of sub-physiological stiffness (1kPa) and supraphysiological stiffness (10, 50 kPa), as well as coated and uncoated glass coverslips as a control. This setup allows variation in stiffness without changing the chemical adhesion components and ensures specific activation of β1-integrins through their known preference for collagen I (Humphries, 2000). To assess polarity, we studied three hallmark characteristics of apical-basal polarization: appropriate localization of apical proteins with species-specific markers, formation of cortical actin and increased cell height (Meder et al., 2005; Bryant and Mostov, 2008; St Johnston and Ahringer, 2010; Shen et al., 2011). Furthermore, we examined the influence of β1-integrin on polarization by studying the distribution of the β1-integrins and by comparing polarization in the presence and absence of a β1-integrin blocking antibody.

## Materials and Methods

### Madin Darby Canine Kidney cells Culture

Madin Darby Canine Kidney II cells (MDCK-II, ECACC, Netherlands) were cultured and expanded in a standard cell culture incubator (37°C, 5% CO_2_) in Eagle’s Minimum Essential Medium (EMEM; Merck, Darmstadt, Germany) supplemented with 5% fetal bovine serum (FBS; Greiner Bio-one, Alphen aan de Rijn, Netherlands), 1% penicillin/streptomycin (Invitrogen, Waltham, MA, United States) and 1% l-glutamine. Culture medium was renewed every 3–4 days and the cells were passaged when reaching 80% confluency. Cells were seeded on polyacrylamide substrates and glass substrates at a density of 5.000 cells cm^−2^ for immunofluorescence analysis. To treat the cells with AIIB2 (University of Iowa, Iowa city, IA, United States), the antibody was added to the medium in a final concentration of 2 µg mL^−1^. Experiments were replicated in triplicate (*n* = 3).

### Tubuloid Culture

Tubuloid cultures were established and cultured as previously described (Gijzen et al., 2021). Experiments were approved by the medical ethical committee of the University Medical Center Utrecht and informed consent was obtained from the patient beforehand. Tubuloids were dissociated to single cells as previously described (Gijzen et al., 2021). Briefly, tubuloids were incubated in 1 mg ml^−1^ dispase II (Thermo Fisher Scientific, Waltham, MA, United States) for 30 min at 37°C to remove basement membrane gel. Tubuloids were then washed, sheared using a flame polished Pasteur pipet and incubated in accutase (Thermo Fisher Scientific, Waltham, MA, United States) with 10 μM Y-27632 Rho-Kinase inhibitor (Abmole, United States) at 37°C. After 45 min, a single cell suspension was achieved. Cells were washed and counted with Trypan Blue. 50.000 tubuloid-derived cells in advanced DMEM-F12 with 1% penicillin/streptomycin, 1% GlutaMax and 1% HEPES (ADMEM-F12+++; all reagents from Thermo Fisher Scientific, Waltham, MA, United States) were plated to 12-wells culture plates containing glass or 1, 10 or 50 kPa polyacrylamide substrates. After timepoints 16, 24, and 48 h and 5 days, tubuloid-derived cells on these different substrates were washed twice, fixed with 4% paraformaldehyde for 30 min, washed twice and stored at 4°C for immunofluorescence analysis. Experiments were replicated in triplicate (*n* = 3).

### Polyacrylamide Substrate Preparation

**FIGURE 1:**
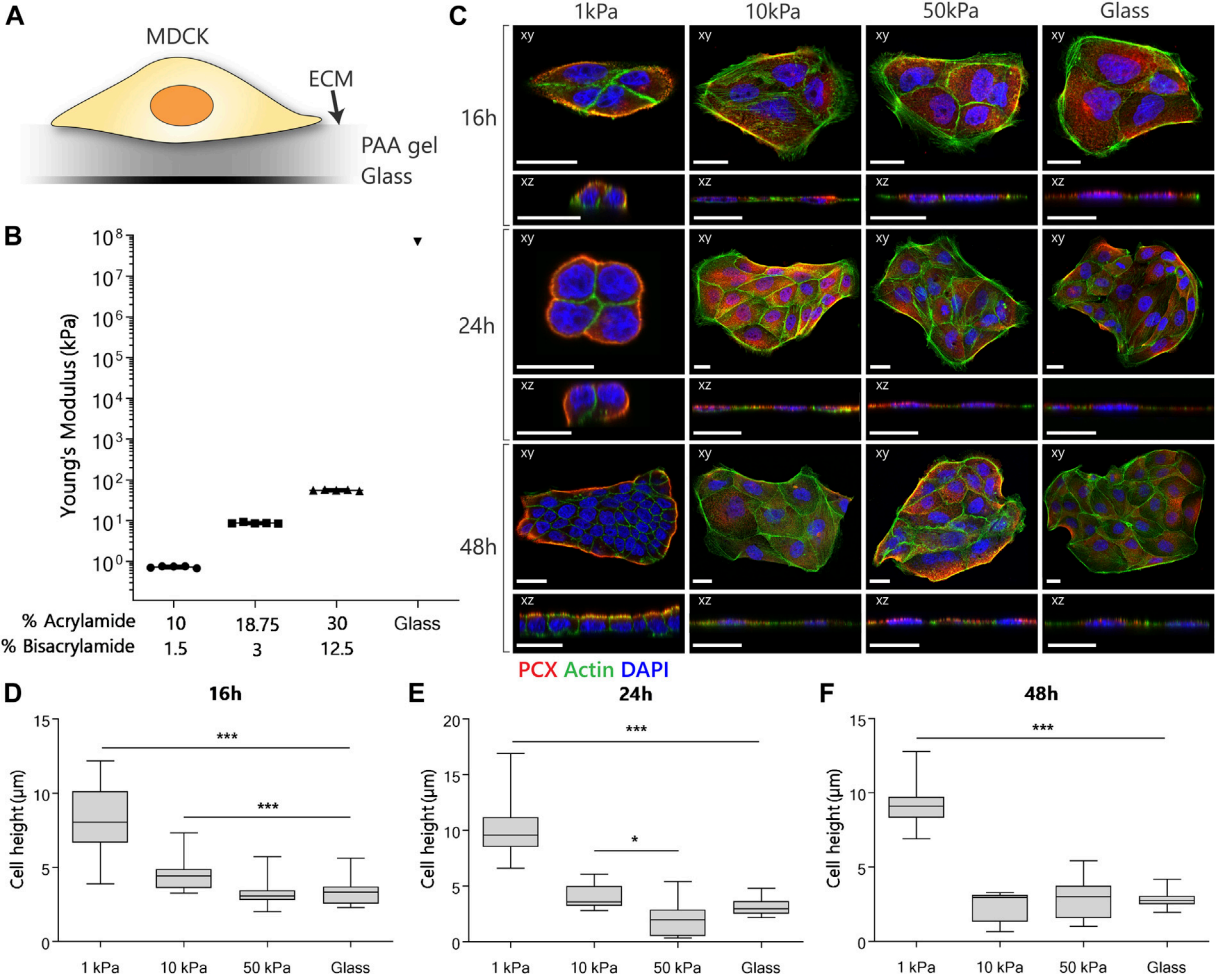
MDCK polarization and morphology when cultured on substrates with different stiffnesses. **(A)** Experimental setup for the examination of the effect of different substrate stiffnesses on the establishment of apical-basal polarization. **(B)** The Young’s modulus of the substrates was verified using nanoindentation. **(C)** Representative fluorescent images of MDCKs cultured on PAA substrates with an elastic modulus of 1, 10 and 50 kPa or on glass controls. The cells were stained for podocalyxin (red), actin (green) and nuclei (blue). Confocal sections are shown in both xy (top) and xz (bottom) direction. Scale bars are 20 µm. (D/E/F) Cell height quantification of MDCKs at the 16-hour **(D)**, 24-hour **(E)** and 48-hour **(F)** time point (*n* = 3 per group) using a dedicated Matlab script. (*p*-values are calculated using a Kruskal-Wallis test with a Dunn’s multiple comparison test, *p < 0.05, **p < 0.01, ***p < 0.001).

One hundred micro meters thick polyacrylamide (PAA) hydrogel culture substrates were fabricated by first functionalizing glass substrates with a 7% bind-silane solution (PlusOne Bind-Silane; Merck, Darmstadt, Germany). Phosphate Buffered Saline (PBS; Merck, Darmstadt, Germany), 40% acrylamide (Bio-Rad, Veenendaal, Netherlands) and 2% bis-acrylamide (Bio-Rad, Veenendaal, Netherlands) were mixed in different weight percentages to obtain the variety in Young’s modulus (Figure 1B). Polymerization was initiated by adding 10% ammonium persulfate (APS; Bio-Rad, Veenendaal, Netherlands) and *N,N,N′,N′*-tetramethylenediamine (TEMED; Merck, Darmstadt, Germany) for a duration of 60 min at room temperature (RT). For cross-linking ECM-proteins to the hydrogel, 150 µl of 1 mg ml^−1^ Sulfosuccinimidyl 6-(4′-azido-2′-nitrophenylamino) hexanoate (Sulfo-SANPAH; Merck, Darmstadt, Germany) was placed on all substrate surfaces and exposed to an ultraviolet (UV) light (Camag, 16W) for 10 min under 366 nm wavelength at 5 cm distance. The substrates (including glass substrates) were then transferred to a sterile culture cabinet and rinsed three times with sterile PBS. Next, 100 µl of a 0.1 mg ml^−1^ rat-tail collagen I solution (Corning, Amsterdam, Netherlands) was placed on top of the substrate and incubated overnight at 4°C. After incubation, the collagen-coated PAA substrates were rinsed thrice with sterile PBS before cell culture. The Young’s modulus of the PAA substrates was quantified using nano-indentation with the PIUMA nano-indenter (Optics11, Amsterdam, Netherlands).

### Immunofluorescence Staining

Cells were fixed with 3.7% formaldehyde solution (Merck, Darmstadt, Germany) in PBS for 15 min at RT after 16, 24 and 48 h post seeding and washed three times with PBS. Cells were subsequently permeabilized with a 0.5% Triton-X-100 (Merck, Darmstadt, Germany) in PBS for 5 min at RT and blocked for 1 h at RT in PBS with 10% horse serum to prevent nonspecific antibody binding. The cells were then stained with the primary antibodies listed in Table 1 overnight at 4°C in PBS with 1% horse serum. After washing three times with PBS, the cells were incubated with secondary antibodies in PBS for 1 h at RT. Cells were washed three times with PBS and subsequently incubated with a 4′-6-diamidino-2-phenylindole solution (DAPI) in PBS. Cells were finally washed three times with PBS and mounted with Mowiol (Merck, Darmstadt, Germany). Images of approximately 10–20 cell clusters per condition were acquired using a Leica SP8 microscope with oil-63x magnification and further analyzed using deconvolution software (Huygens, Hilversum, Netherlands).

**TABLE 1:** List of used antibodies and dyes. Abbreviations used in this table: SA, Sigma Aldrich; BD, Biosciences; MI, Merck Millipore; GT, Gene-Tex; TF, Thermo-Scientific.

Antigen	Source	Cat. No	Isotype	Label	Species	Dilution IF
Podocalyxin	MI	3073161	IgG1	A555	Mouse	1/200
β1-integrin	GT	GTX128839	IgG	A555/A488	Rabbit	1/200
Crumbs3	TF	PA5-53092	IgG	A555	Rabbit	1/100
Zona Occludens 1	BD	610,966	IgG1	A488	Mouse	1/200
Vinculin (MDCK)	SA	V9131	IgG	A555	Mouse	1/200
Vinculin (Tubuloid)	TF	42H89L44	IgG	A555	Rabbit	1/200
Phalloidin	SA	P1951		A488/A647	Amanita phalloides	1/200
DAPI	SA	D9542				1/500

### Cell Height Quantification

The cell height was determined from the immunofluorescence confocal images using a custom-built Matlab script (version 2019b, Mathworks Inc., Portola Valley, CA, United States). Briefly, the height of 2-3 cells per cluster was determined as the distance between the base of the cell and its highest point. These distances were then averaged to determine one height per cluster. Per condition in each of the replicates approximately 10–20 cell clusters were selected and analyzed.

### Statistical Analysis

All data are represented as mean ± standard deviation. Data were tested for normality using the Shapiro Wilk test. Since a part of the experimental groups was not normally distributed, a Kruskal Wallis test followed by a Dunnett’s Multiple Comparison Test was subsequently performed to identify statistically significant differences (GraphPad, La Jolla, CA, United States) between the experimental groups and the glass control group. Differences were considered as statistically significant when *p* < 0.05.

## Results and Discussion

### The Establishment of Apical-Basal Polarization in MDCKs Is Mechanosensitive

To investigate the influence of substrate stiffness on apical-basal polarization in a 2D *in vitro* model, we cultured MDCKs on collagen-I coated substrates with different elastic moduli (Figure 1A). Cell clusters were cultured on substrates of 1, 10 and 50 kPa (Figure 1B), representing sub-physiological (1 kPa) and supraphysiological (10 and 50 kPa) stiffnesses (Handorf et al., 2015). Collagen-I coated, and uncoated glass cover slips were used as control. The substrates of 1 kPa, below the physiological stiffness in the kidney, were shown to induce a cuboidal morphology with distinct apical basal polarization in MDCKs. The MDCKs cultured on the substrates above the physiological stiffness of the kidney (10 kPa, 50 kPa PAA substrates and the glass coverslips) did not portray any of the polarization characteristics.

To characterize apical-basal polarization, we stained for podocalyxin (PCX) as a widely used canine apical membrane protein marker in MDCKs (Ojakian and Schwimmer, 1988; Cheng et al., 2005), and the actin cytoskeleton with phalloidin and visualized these markers using confocal microscopy. From the confocal images we quantified cell height using a dedicated Matlab script. On the 1 kPa substrates, PCX localized exclusively to the boundaries of the cell clusters after 24 h (Figure 1C). Furthermore, MDCKs displayed cortical actin organization and an increased cell height (Figures 1D,E,F) on 1 kPa substrates. The same response was observed after 48 h in cell clusters with an increased number of cells due to proliferation, indicating stabilization of polarization. In the case of the 10 and 50 kPa substrates, and the glass control, MDCKs exhibited evenly dispensed PCX in the cytoplasm throughout the entire culture (Figure 1C), together with a flat cell morphology (Figures 1D,E,F). An increase in the formation of actin filaments, cortical and ventral, was observed in all cells grown on the stiffer substrates. Finally, no difference in alle polarity readouts was observed between cells cultured on coated and uncoated glass substrates (Supplementary Figure S1).

Other studies that grow MDCKs on different 2D substrates report inconsistent findings with respect to each other. Kaliman et al. (2014) also investigated the difference in morphology and proliferation for MDCKs grown on collagen-I coated 0.6 kPa PAA substrates compared to glass coverslips and found the same difference in morphological characteristics compared to our study. However, the establishment of apical-basal polarization was not investigated immunohistochemically (Kaliman et al., 2014). In contrast, the study by Mrozowska and Fukuda. reported that MDCKs were able to adopt a cuboidal morphology and polarize in 48 h on uncoated glass (Mrozowska and Fukuda, 2016). Studies using porous supports, such as transwell filters [∼3 GPa (Larsson and Dérand, 2002)], have shown MDCKs with a cuboidal morphology and a localized expression of polarization markers. The enhanced differentiation and apical-basal polarization of cells grown on porous supports compared to non-porous supports was explained by the access to nutrients at the basolateral surface. However, in the studies involving porous supports the role of stiffness was not investigated (Lipschutz et al., 2001; Vinaiphat et al., 2018).

### The Mechanosensitivity of Apical-Basal Polarization in MDCKs Is Mediated by β1-Integrins

**FIGURE 2:**
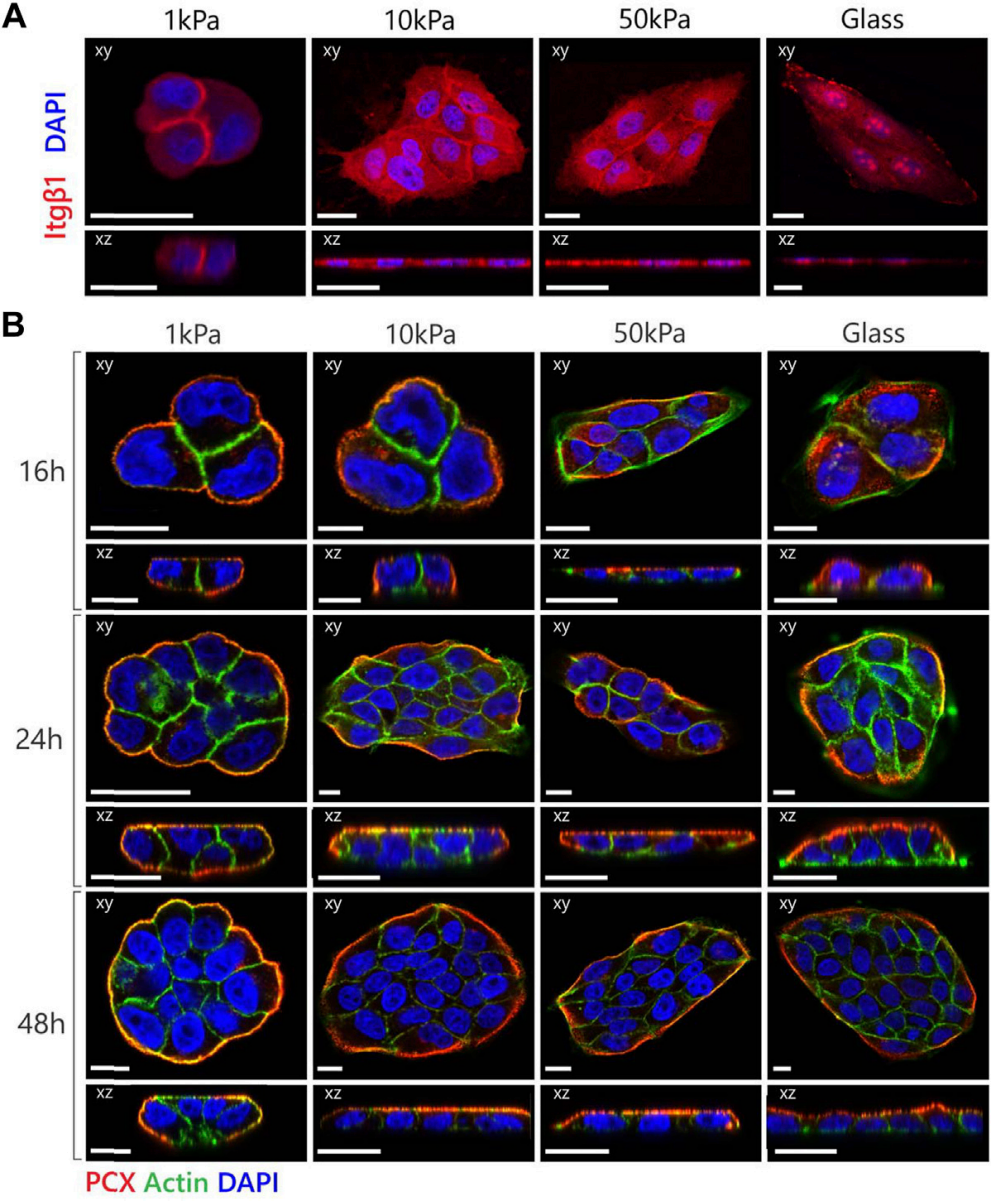
MDCK polarization is dependent on expression as well as location of the β1-integrin receptor. **(A)** Representative fluorescent images of β1-integrin expression in MDCKs cultured on substrates with variable elastic moduli. The cells were stained for β1-integrin (red) and nucleus (blue). **(B)** Representative fluorescent images of MDCK cells cultured on substrates with variable elastic moduli with the addition of the β1-integrin inhibitor AIIB2 to the culture medium in a final concentration of 2 µg mL^−1^. The cells were stained for podocalyxin (red), actin (green) and nuclei (blue). Confocal sections are shown in both xy (top) and xz (bottom) direction. Scale bars are 20 µm.

β1-integrins are known to be crucial mediators of polarization as disruptions of the function of β1-integrins in MDCKs led to the prevention of cyst polarization in a 3D environment (Ojakian and Schwimmer, 1994; Yu et al., 2005; Taddei et al., 2008; Myllymäki et al., 2011; Akhtar and Streuli, 2013). To understand the role of β1-integrins in the substrate stiffness-dependent establishment of apical-basal polarization, we examined the localization of β1-integrins within the MDCKs on different substrates (Figure 2A). The integrins at the sub-physiological substrates were localized primarily on the cell-cell boundary, while the integrins at the supraphysiological substrates were more diffuse throughout the cytoplasm. When cultured on glass substrates, the cells also exhibited clear clustering of the β1-integrins. Together with the difference in morphology, the location and clustering of the integrins suggests that the sub-physiological substrates allow diminished cell adhesion that allows the adoption of a cuboidal morphology necessary for the establishment of polarization.

Based on these findings, we inhibited integrin (adhesion) functionality by adding the AIIB2 inhibitory antibody to the medium in a final concentration of 2 μg ml^−1^ during culture. We observed that MDCKs on supraphysiological substrates were now able to polarize within 24 h (Figure 2B). In the MDCKs grown on 1 and 10 kPa substrates, PCX localized immediately at the boundaries of the cell cluster (Figure 2B—16 h). Combined with the cortical actin organization and the increase in cell height, the cells displayed clear polarization characteristics relatively early. The MDCKs on the 50 kPa substrates and the glass coverslips also showed PCX localization at the boundaries of the cell cluster, although at a later time point (Figure 2B—24 h). Also, at this moment in time, only cortical actin was present in the cell clusters and no change in cell height was measured. While the MDCKs on the 10 and 50 kPa and glass substrates had the same morphological and polarization characteristics over time with an increase in cell number due to proliferation, the cells on the 1 kPa substrates displayed vertical proliferation (on top of each other) instead of horizontal proliferation (monolayer formation) (Figure 2B—48 h).

In contrast, studies in 3D cultures have demonstrated that inhibition of β1-integrins prevented polarization. We hypothesize that the difference in response to the β1-integrin inhibition between 2D and 3D environments can be explained by opposing roles of β1-integrins in the polarization process. On the one hand, the signaling cascade *via* the integrin-receptors is necessary for the initiation of apical-basal polarization (Wang et al., 1990a; Wang et al., 1990b; Ojakian and Schwimmer, 1994; Yu et al., 2005; Akhtar and Streuli, 2013). On the other hand, the anchorage of integrins to the substrate, which is increased with increasing substrate stiffness (Gallant et al., 2005), is associated with a spreading morphology of the cells that creates an inhibitory effect to polarization. In our experimental set-up, the increased cellular adhesion of the cells grown on supraphysiological substrates may be dominant over the integrin-mediated signaling pathway involved in apical-basal polarization. In the 3D culture, the inhibitory effect on polarization by the spreading morphology is not present, presumably due to the differences between 2D and 3D cellular adhesion (Harunaga and Yamada, 2011). Our hypothesis concerning the competing role of the β1-integrins suggests that a different strategy may be required for successfully establishing polarization in the bio-artificial kidney (2D) compared to tissue-engineered kidney tubules (3D).

### Tubuloid-Derived Cells Do Not Express All Hallmarks of Apical-Basal Polarization and Are Not Mechanosensitive

**FIGURE 3:**
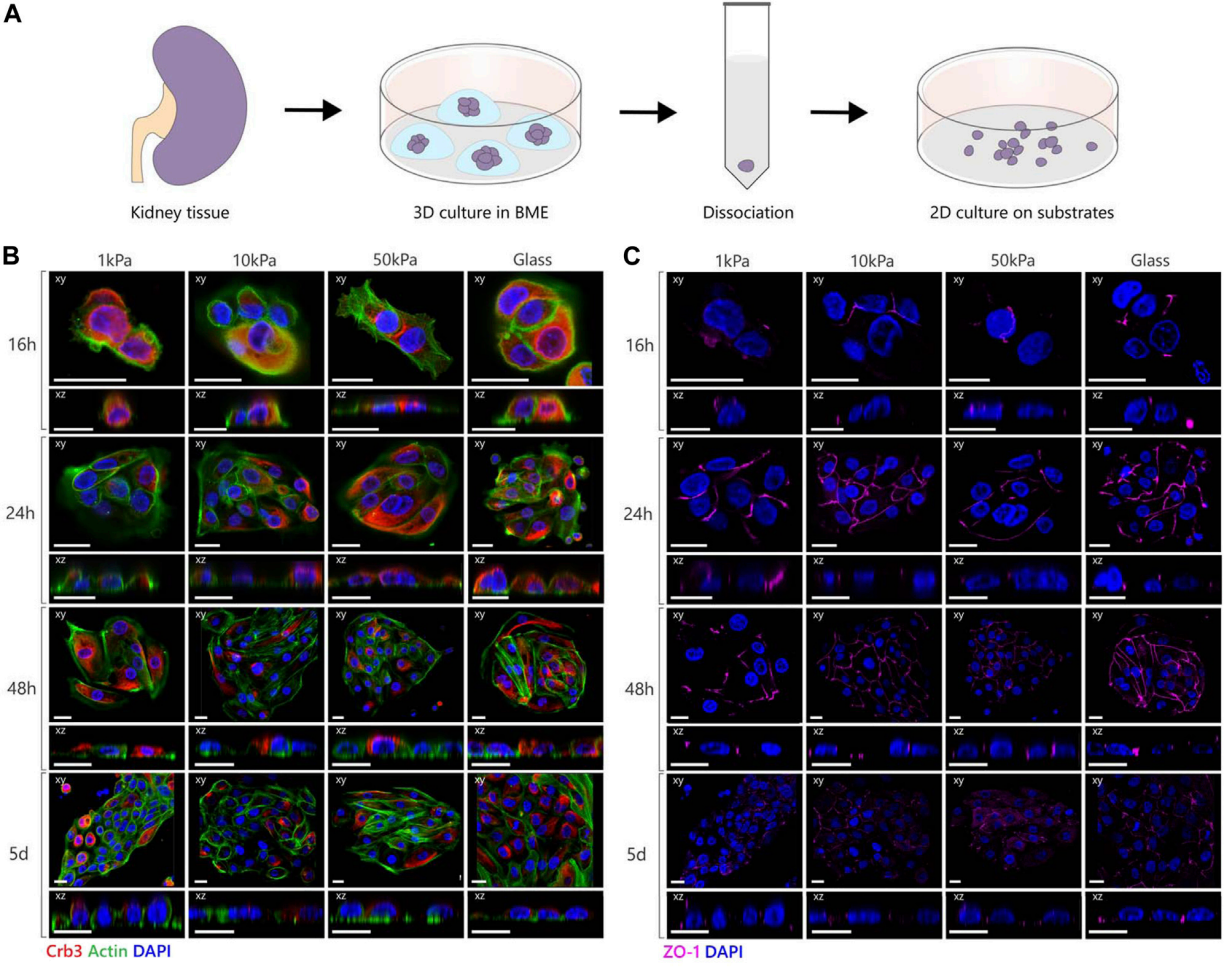
Polarization and junction formation of tubuloid-derived cells on substrates with different elastic moduli. **(A)** Schematic image depicting the culture of tubuloid-derived cells isolated from cortical human kidney. **(B,C)** Representative fluorescent images of tubuloid-derived cells cultured on PAA substrates with an elastic modulus of 1, 10 and 50 kPa or on glass substrates. The cells were stained for either Crb3 (red), actin (green) and nuclei (blue) or for Zona Occludens 1 (magenta) and nuclei (blue). Confocal sections are shown in both xy (top) and xz (bottom) direction. Scale bars are 20 µm.

Next, we seeded tubuloid-derived cells that are established from primary human kidney epithelium on the different 2D substrates to investigate if the polarization mechanism found in MDCKs translates to primary human epithelial cells. (Figure 3A) (Schutgens et al., 2019; Yousef Yengej et al., 2020; Gijzen et al., 2021). Since PCX is in humans mainly expressed by podocytes and thus not an appropriate interspecies marker for cellular polarization (Uhlén et al., 2015), we stained for the apical protein Crumbs3 (Crb3) and tight-junction associated protein Zona Occludens 1 (ZO-1) as specific markers for apical-basal polarization in human epithelial cells. Crb3 and ZO-1 were expressed by all tubuloid cell clusters throughout the culture thereby displaying the epithelial nature of these tubuloid-derived cells (Figures 3B,C). However, distinct apical localization of Crb3 was absent in the tubuloid-derived cells on all substrates during the entire duration of the culture. The tight junctions, as marked by ZO-1, were arranged both apically as well as basally along the lateral membrane in all conditions, instead of localizing solely at the apical side of the lateral membrane as is the case in physiological epithelial polarization (Balkovetz, 2009). The tubuloid-derived cells did display the cuboidal morphology that was expected for polarized epithelial cells. Also, the organization of the actin cytoskeleton remained unchanged during the culture period, with F-actin fibers at the base of the cell and a distinct actin cell cortex. We did not observe any differences between different patches of tubuloid cells, indicating similar responses across the different tubular cell types.

**FIGURE 4:**
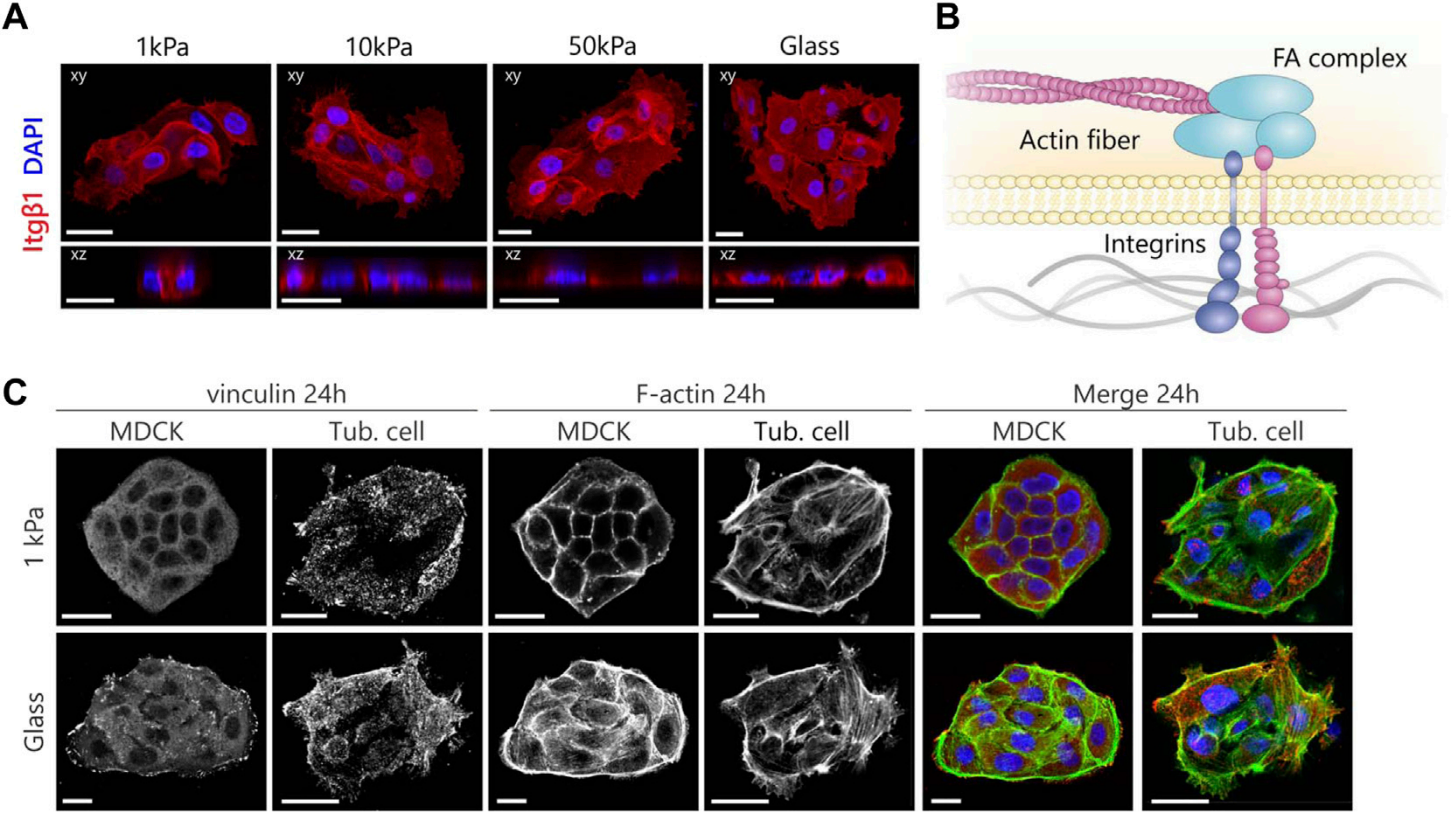
MDCKs and tubuloid-derived cells show a different organization of the mechanosensing machinery according to substrate stiffness. **(A)** Representative fluorescent images of β1 integrin expression in tubuloid-derived cells cultured on substrates for 24 h with variable elastic moduli. Cells were stained for β1 integrin (red) and nuclei (blue). **(B)** A graphical illustration displaying the mechanotransduction pathway that acts via the dynamic link the FA complex forms between integrins and the actin cytoskeleton. **(C)** Immunofluorescence images displaying the differences in maturation and organization of the focal adhesions in red (vinculin) and the cytoskeleton in green (F-actin) after 24 h. Scale bars are 20 µm.

To shine a light on the contrast between the observed characteristics of polarization, we again investigated the localization of β1-integrins (Figure 4A). The cells showed no distinct variations in localization of β1-integrins as a function of substrate stiffness. The integrins were diffuse throughout the cytoplasm without showing any clustering on the cell-matrix boundary but did show some localization to the cell-cell boundaries. The presence of β1-integrins in the tubuloid-derived cells indicates that the cells could polarize *via* the integrin-mediated signaling pathway. Additionally, both the cuboidal morphology and the cortical organization of the cytoskeleton suggest that the inhibitory effect posed by increased cellular adhesion is not present in the tubuloid-derived cells, and therefore cannot explain the random localization of both the apical proteins and the tight junctions. These observations combined suggest that other factors could be responsible for the establishment of apical-basal polarization in tubuloid-derived cells on 2D substrates. We could argue that the studied markers for the different cell types complicate the comparison, especially since the MDCKs are distal tubular epithelial cells and the tubuloids contain epithelial cells from other segments including loop of Henle and collecting duct. Additionally, these stem cell derived cells are suggested to function better in a 3D environment, as the tubuloids in 3D culture were previously reported to contain cuboidal epithelium with a cortical actin cytoskeleton and display polarized expression of cilia and functional transport proteins (Schutgens et al., 2019; Gijzen et al., 2021). In this 3D culture, the cells were cultured in basement membrane gels that consist of multiple ECM components such as collagen I, collagen IV, and several laminins. These basement membrane gels thus more closely resemble the physiological architecture and composition of the tubular ECM. The inaccurate replication of the *in vivo* environment by the 2D culture has already been proven nonbeneficial for stem cells (Lv et al., 2017) and this could also be the case for the tubuloid-derived cells. Furthermore, in this study we chose a collagen-I coating as we specifically focused on the known link between the β1-integrin and apical-basal polarization *via* Rac1 (O’Brien et al., 2001; Yu et al., 2005). However, apical-basal polarization could be mediated by different integrin heterodimers as well. For instance, while collagen activates Rac1 *via* α1β1, α2β1 or α11β1, laminin is inclined to activate α3β1 and α6β1. Myllymäki et al. (2011) demonstrated that in 3D collagen-I gels α2β1- and β4-integrins are necessary for the induction of polarization and the subsequent lumen formation, while in BME gels the activation of α3β1 was required. Additionally, the duration of the culture could also be a factor as for lung epithelial cells, Szymaniak et al. (2015) showed that polarization of progenitor cells isolated from mouse tracheae emerged after 10 days. Therefore, future studies should further explore the effects of different physiologically relevant ECM coatings (e.g., laminins, collagen IV) as well as culture duration, and extend the experimental setup to 3D to more closely resemble the *in vivo* situation.

Moreover, the adoption of the same cellular morphology and cortical organization of the cytoskeleton irrespective of substrate stiffness implies that the tubuloid-derived cells might not be able to sense mechanical cues. To understand this, we further explored the mechanotransduction pathway that links integrins to FA complexes and the cytoskeleton to enable cellular responses to mechanical cues from the environment (Figure 4B). We stained for vinculin as an important FA protein and again for actin using phalloidin to visualize the link to the actin cytoskeleton (Figure 4C). In the case of MDCKs grown on glass, large prominent clusters of vinculin were present throughout the cells and actin fibers were organized in thick bundles connecting to the FA clusters. This organization was different in MDCKs grown on 1 kPa substrates, where vinculin was evenly distributed over the cytoplasm and the cells expressed a cortical cytoskeleton with only a limited presence of basal actin. The adaption of the organization of both the FAs and the actin cytoskeleton to different stiffnesses shows that the MDCKs are able to sense 2D substrate stiffness and are able to respond accordingly. For the tubuloid-derived cells, no differences in their distribution of vinculin and actin fibers in response to changes in 2D substrate stiffness were observed. Vinculin was distributed in small clusters over the entire cell in all cases and a large fraction of these clusters did not colocalize with the actin fibers. Additionally, the actin cytoskeleton was organized in a typical stem cell like fashion with a distinct actin cortex and a high amount of basal actin (Supplementary Figure S2) (Evans et al., 2009; Boraas et al., 2016). The organization of the mechanosensing machinery did not change over 5 days in the tubuloid-derived cells on both substrate stiffnesses (Supplementary Figure S3). The missing connection between the FAs and the actin cytoskeleton could reflect the presence of adult renal stem/progenitor cells in the tubuloid culture, as the seen organization of the cytoskeleton (Supplementary Figure S2) and immature focal adhesions resemble those in progenitor cells in the heart (Mauretti et al., 2016). Unfortunately, we could not compare to progenitor epithelial cells as to our knowledge no studies dedicated to the mechanosensing machinery in stem cell derived epithelial cells have been performed.

## Conclusion

In summary, we discovered that MDCKs established apical-basal polarization on sub-physiological (1 kPa), but not on supraphysiological (>10 kPa) substrates. The ability of MDCKs to polarize is found to not only depend on the known biochemical signaling cascade *via* β1-integrins, but in the case of increased substrate stiffness can be overruled by β1-integrin mediated adhesion of the cell to the substrate. Both soft substrates and inhibition of the β1-integrin eliminates the adhesion that competes with polarization. Human tubuloid-derived cells on the other hand did not fully polarize on any of the investigated 2D substrate stiffnesses but displayed the same cuboidal morphology and cortical actin cytoskeleton organization on all substrates suggesting a diminished mechanosensing capacity. Further analysis indeed showed immature FAs of which a portion was not connected to the cytoskeleton. In summary, this study shows that polarization is a complex process, where cell type, extracellular environment, and the chemical and mechanical aspects of integrin mediated cell-matrix interactions play a role. Further exploring the underlying mechanism in polarization to understand the different factors will facilitate the progression of developing strategies for both the bio-artificial kidney as well as kidney tissue engineering. Future studies should also include the extension towards a 3D *in vitro* model to investigate if the effects of polarization to matrix stiffness are comparable to 2D and how this knowledge could be applied to direct differentiation, polarization, and cellular organization in bioengineered and/or bioartificial kidneys.

## Data Availability

Orginial data underpinning the conclusions presented in this work are included in the article/Supplementary Material. Any reasonable requests for raw data can be directed to the corresponding author.
