# Supplementary figures and images for: Substrate Stiffness Determines the Establishment of Apical-Basal Polarization in Renal Epithelial Cells but Not in Tubuloid-Derived Cells
Source: Front Bioeng Biotechnol. 2022 Mar 1;10:820930. doi: 10.3389/fbioe.2022.820930 (PMC8923587; doi:10.3389/fbioe.2022.820930)

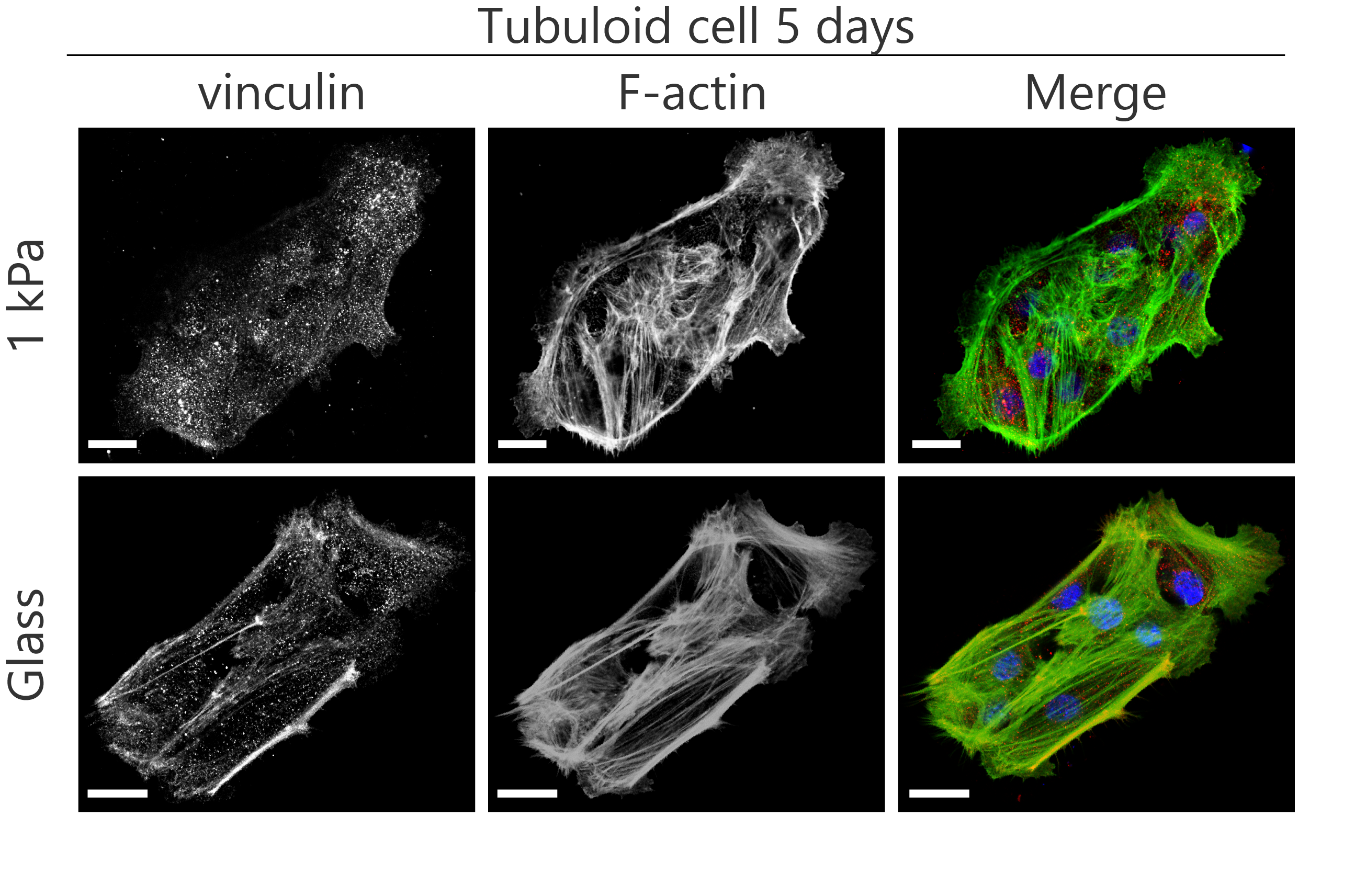

Supplement: Supplementary file 1 [file Image3.TIF]

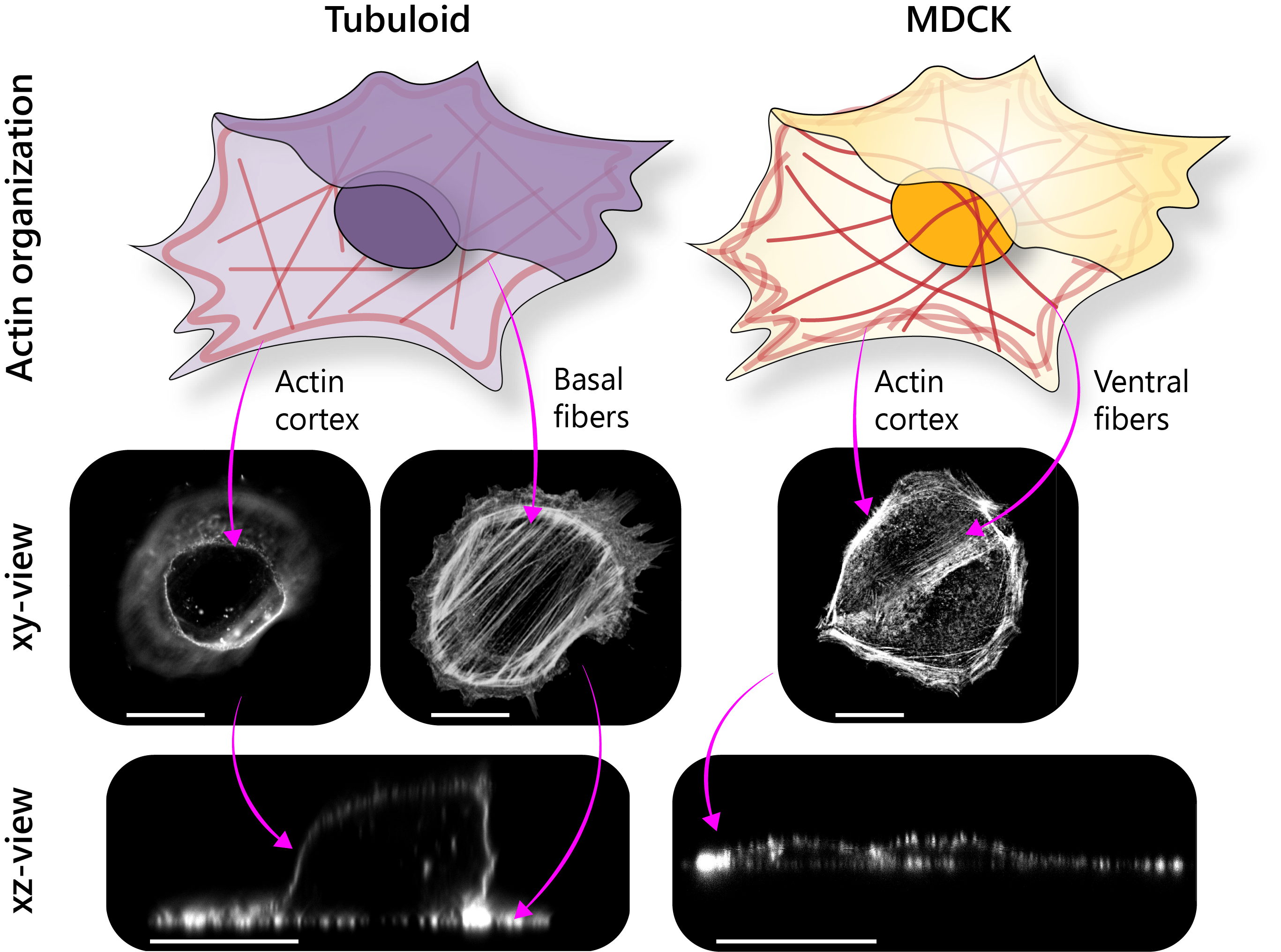

Supplement: Supplementary file 2 [file Image2.TIF]

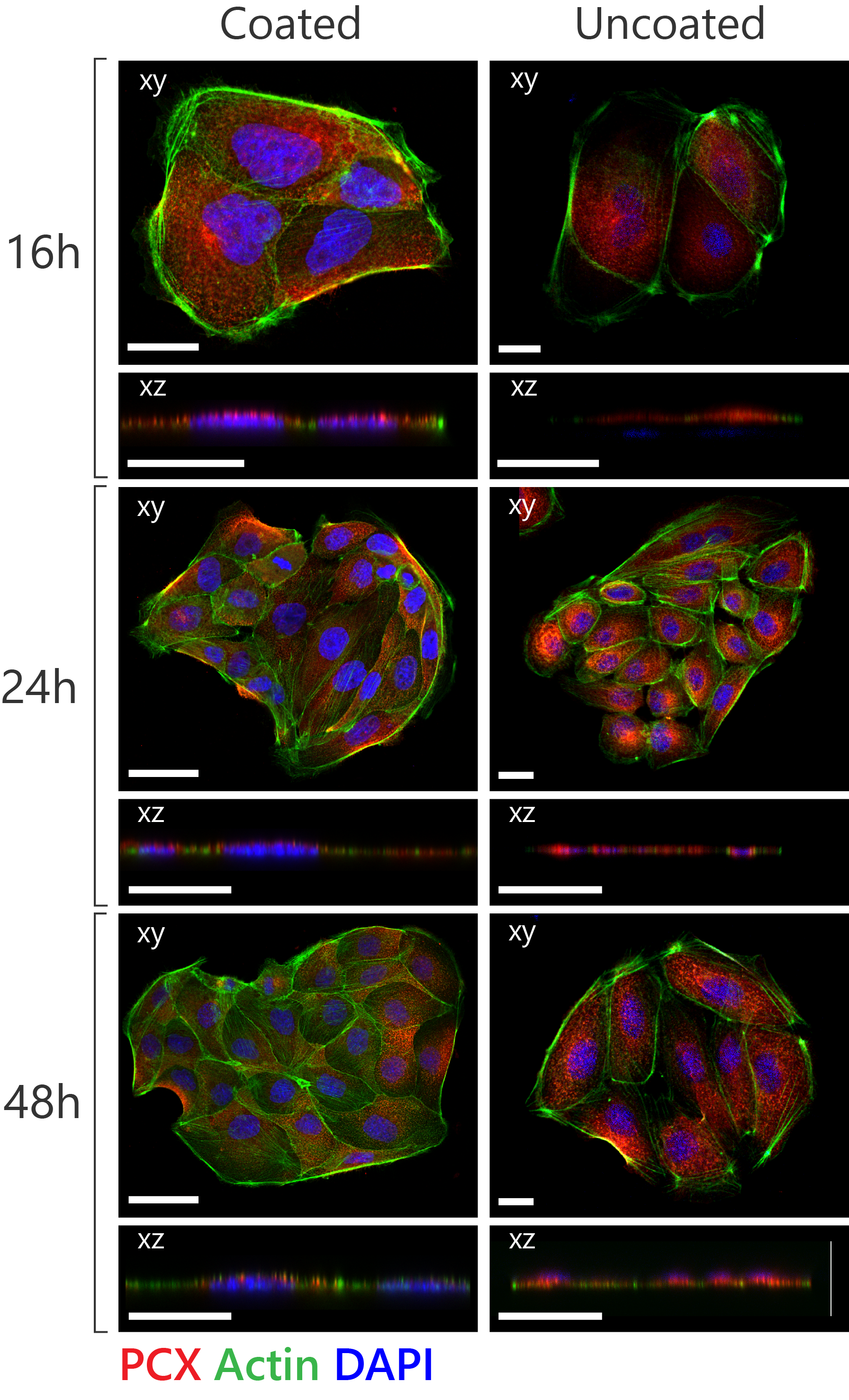

Supplement: Supplementary file 3 [file Image1.TIF]
