# Supplementary material for: Substrate Stiffness Determines the Establishment of Apical-Basal Polarization in Renal Epithelial Cells but Not in Tubuloid-Derived Cells
Source: Front Bioeng Biotechnol. 2022 Mar 1;10:820930. doi: 10.3389/fbioe.2022.820930 (PMC8923587; doi:10.3389/fbioe.2022.820930)
Supplement: Supplementary file 4 [file DataSheet1.pdf]

Supplementary Figure Legends

Supplementary Figure 1. Representative fluorescent images of MDCK cells cultured on uncoated and coated glass substrates. The cells were fixed with PFA at the times indicated and then stained for podocalyxin (red), actin (green) and the nucleus (blue). Confocal sections are shown in both xy (top) and xz (bottom) direction. Scale bars are 20 µm.

Supplementary Figure 2. Schematic illustration highlighting the differences in organization of the actin cytoskeleton between MDCKs and tubuloid cells when grown on glass coverslips. Cross-sections of the MDCKs and tubuloid cells show the different types of actin organization: actin cortex, ventral fibers, and basal fibers. The actin cytoskeleton of the MDCKs contains more fibers that have a variable thickness, with also fibers visible that cross over the nucleus (the ventral fibers). The tubuloid cells on the other hand have a more stem cell-like organization with a thin cortex and basal actin at the bottom of the cell. Scale bars are 20 µm.

Supplementary Figure 3. Immunofluorescence images displaying the differences in maturation and organization of the focal adhesions in red (vinculin) and the cytoskeleton in green (F-actin) after 5 days. Scale bars are 20 µm.
